# Minutes of the Third Regular Meeting of the Western Dental Society

**Published:** 1857-07

**Authors:** 


### PROCEEDINGS OF SOCIETIES.

### ARTICLE VIII.

### *Minutes of the Third Regular Meeting of the Western Dental Society.*

St. Louis, *May* 2, 1857.

The Society met as per adjournment, in the Temperance Hall, at 10½ o'clock, and was called to order by the President. An abstract of the minutes of the last meeting, held at Chicago, was read by the Secretary.

The Treasurer made the following report, as the state of his account, and requests the auditing committee to examine the same:

| | |
|---|---|
| Received for membership, . . . . . | $185 00 |
| The disbursements for various purposes amount to | 126 06 |
| Leaving a balance in the treasury of, | $ 58 94 |

All of which is respectfully submitted,

A. Blake, *Treas.*

Which on motion was received.

The accounts of the Treasurer not having been audited, the above motion was reconsidered, and the account referred.

On motion of Dr. Allport, Dr. Taft was elected an honorary member of this society.

Dr. Allport moved that a business committee of three be appointed, which was carried, and Drs. Allport, Spalding and Peebles were appointed said committee.

On motion of Dr. Spalding, a recess was taken to give the committee time to report.

After the time had expired, the meeting was called to order, and the committee reported business for the action of the society, as follows:

*Report of Business Committee.*

I. Election of members.

II. Election of officers for the ensuing year.

III. Miscellaneous business.

1st. The best method of arresting decay in the teeth.

2d. Causes and treatment of diseased gums.

3d. Cheoplasty.

4th. Difficult dentition.

5th. In what cases, if any, is it answerable to preserve the fangs of the natural teeth for the reception of artificial crowns.

The committee reported the following gentlemen as having passed satisfactory examinations, and recommended them for membership: Dr. Hubbard, of Quincy, Ill., Drs. A. M. Leslie, C. B. Porter, D. L. Levett, of St. Louis, who were unanimously elected.

On motion it was decided to proceed to the election of officers in the usual way.

Pending the election, it was, on motion, ordered that a committee of two be appointed to invite dentists residing in the city, and who are not members, and other members of the profession who might be in the city, to attend upon the deliberations of the society. Drs. Silvers and Leslie were appointed a committee.

On motion of Dr. Spalding, it was *Resolved*, That the officers be elected by an informal ballot, which resulted as follows:

*President*—Dr. Isaiah Forbes.

*First Vice-president*—Dr. H. N. Lewis.

*Second do.* Dr. C. B. Porter.

*Recording Secretary*—Dr. H. E. Peebles.

*Corresponding Secretary*—Dr. H. J. B. McKellops.

*Treasurer*—Dr. A. Blake.

*Executive Committee*—Drs. H. Barron, W. W. Allport, S. Dunham, E. Hale, Sen., and —— Bull.

Dr. Hale, the former President, conducted the President elect to the chair, who delivered a very appropriate address for the occasion.

On motion of Dr. Perrine, the thanks of this society were tendered Dr. Hale, for the ability with which he has conducted the proceedings of the society for the last year.

Dr. Allport moved that the chair appoint five delegates to the American Dental Convention, to meet in Boston in August next.

The society adjourned to meet at 3 o'clock, P. M.

*Afternoon Session.*—In pursuance of adjournment, the society met at 3 o'clock, P. M.

President in the chair. The minutes of the morning session were read and adopted.

Reports of committees being called for, the committee on by-laws reported as follows:

BY-LAWS.

*Article* 1. The constitution of the W. D. Society shall be deemed the fundamental law of this society.

*Article* 2. This society shall be governed by parliamentary usage.

ORDER OF BUSINESS.

*Article* 3. 1st. The minutes of the preceding meeting shall be read. 2d. Reports from Treasurer and standing committees. 3d. Election of members. 4th. Election of officers. 5th. Unfinished business. 6th. Miscellaneous business. 7th. Reading essays and papers. 8th. Payment of dues. 9th. President's address. 10th. Reading, correcting and adopting the minutes.

*Article* 4. No member shall speak more than once on the same subject, till all have had an opportunity to speak.

All of which is respectfully submitted.

W. W. ALLPORT,<br>H. E. PEEBLES,<br>I. FORBES, } *Committee.*

On motion of Dr. Spalding, the report was accepted. The By-Laws were then read, amended and adopted article by article.

Miscellaneous business being then in order, Dr. Perrine offered a preamble and resolution on local societies.

WHEREAS, It is evident to the observing dentist that our profession has been more rapidly advancing in usefulness and knowledge since the organization of the American Dental Society than at any former period of time. And, whereas, this fact is mainly attributable to organized association and its concomitants, the general and firm interchange of sentiment and diffusion of knowledge, through free discussion, the school and the press. And, whereas, the multiplication of dental societies in our country has greatly increased the facilities for acquiring correct information, and emulating individual enterprise. Therefore,

*Resolved,* That we approbate, encourage and advise the formation of local societies in all places where they can be sustained.

The motion was seconded by Dr. Allport, and adopted.

Dr. Allport moved that a committee of five be appointed, with Dr. Taft as its chairman, to examine, test and report on "cheoplasty." Adopted.

The President appointed Drs. Taft, Allport, Lewis, McKellops, Leslie and Dunham as said committee.

Dr. Perrine moved that a committee of three be appointed to consider and report the subject of patenting by the dental profession.

Drs. Perrine, Barron and Hubbard were appointed. No more miscellaneous business being presented, the regular order of business was taken up.

The best manner of arresting decay in teeth was then discussed.

On motion of Dr. Barron the Secretary called the roll, and each member present responded, and gave his views upon some special case of filling, filing or cutting, and polishing, aided by diagrams on blackboard.

Adjourned to meet at 8 o'clock, P. M., in room of St. Louis Dental Society; and also to meet in this Hall at 9 A. M., tomorrow.

*Evening Session.*

Pursuant to adjournment, the society met in the rooms of the St. Louis Dental Society, at 8 P. M. Second Vice-President, Porter, in the chair.

Discussion on tooth filling continued, which took rather a wide range, embracing collateral issues.

Dr. Perrine presented the name of Dr. I. B. Branch, of Galena, Ill., for membership.

Adjourned to meet at the Hall at 9 A. M., to-morrow morning.

*Second Day—Morning Session.*

Pursuant to adjournment, the society met in their Hall, at 9 o'clock, A. M. President in the chair.

The minutes of the afternoon and evening sessions were read and adopted.

Dr. Geo. H. Silvers presented the name of J. W. Fulton, of Paris, Ill.; Dr. E. Hale, Jr., presented the name of Dr. McCuen, of Louisiana, Mo., and Dr. Perrine presented the names of Dr. A. Blakesly, of Utica, N. Y., and Dr. C. M. Forbes, as candidates for membership.

*Miscellaneous Business.*—Dr. Perrine offered the following resolution:

*Resolved*, That all members present having performed any operation requiring peculiar manipulation, instruments or anything new, may have the opportunity to present them after the order of business.

After which Dr. I. B. Branch, of Galena, Ill., was elected an active member, and Dr. A. Blakesly, of Utica, N. Y., an honorary member.

Dr. Spalding received a letter from Dr. A. W. French, of Springfield, Ill.

On motion of Dr. Perrine, the letter was read, and the following resolution adopted.

*Resolved*, That the exhibition of specimen cases of dental mechanism at Cattle Shows, Mechanics' Fairs, and other similar places, can but be regarded as derogatory to our profession, and will never be resorted to by any intelligent dentist who desires its elevation.

After which the discussion on the cause and treatment of diseased gums was resumed. The roll was called, and each member responded, making the discussion interesting, and somewhat amusing.

On motion of Dr. Allport, the discussion of this subject was discontinued after the remarks of Dr. E. Hale, Sen.

The executive committee presented the name of C. M. Forbes for membership, who on ballot was elected.

On motion of Dr. Spalding, a committee of five was appointed on dental fees, to report to-morrow. Drs. Spalding, Allport, Lewis, Branch and Porter were appointed said committee.

On motion of Dr. Allport, the hours from 3 to 5 o'clock, this afternoon, were appropriated to speaking on any or all subjects proper to come up.

Adjourned to meet at 3 o'clock, P. M.

*Second Day—Afternoon Session.*

Met agreeable to adjournment, at 3, P. M.

1st Vice-President in the chair. On motion, the reading of the minutes of the morning session was dispensed with.

On motion of Dr. Dunham, the members were limited to ten minutes.

After this discussion, the society proceeded to the consideration of instruments, apparatus, &c.

Dr. I. Forbes, exhibited an instrument for drying out partially filled cavities. Dr. E. Hale, Jr., presented an instrument used with advantage in swaging plates. Dr. Perrine exhibited a flask for holding a case of teeth while soldering, and, also, a book for recording dental operations. Dr. Peebles, presented an instrument for holding a polishing stone in finishing fillings.

The time having expired for miscellaneous discussions, on motion, it was extended half an hour. And when this had expired the discussion was further extended.

Adjourned to 9 o'clock, A. M., to-morrow.

*Third Day—Morning Session, Friday, 22.*

Meeting called to order, pursuant to adjournment, by the 2d Vice-President, Dr. Porter.

The minutes of the previous session were read. Miscellaneous business was then taken up. Dr. Perrine offered a preamble and resolutions on the death of the late Dr. Hullihen.

WHEREAS, By the inscrutable decrees of Divine Providence, there has been taken from his career of usefulness on earth, to that bourne from whence no traveler returns, Dr. Hullihen, of Wheeling, who by his superior skill and attainments, had become a beacon light in his profession, who by his usefulness and generous manifestation, had not only endeared himself to those whom his skill had benefitted, but to every member of his profession, and which will be embedded in their grateful hearts, until time shall be no more. Had become a radiant star in the bright galaxy of surgical dentistry, ever maintaining that dignity in his profession which enabled him to compel others of a kindred profession to submit to his performing every operation that rightly belonged to dental surgery. Therefore, be it

*Resolved*, That the Western Dental Society, deeply sympathize with the relations of our brother, so suddenly taken in the bloom of life.

*Resolved*, That a copy of these resolutions be transmitted, through the Secretary, to the relatives of the deceased.

*Resolved*, That these resolutions be published in all the dental publications in the Union.

The name of Dr. Hannaman, of Shelbyville, Ill., was presented by Dr. Sturges for membership, and was referred to the executive committee.

The subject of "difficult dentition" was called up, but no discussion elicited.

Reports of special committees were called for, and the committee on dental patents reported, which was received, and after some discussion part of it was laid on the table, and the balance referred back to said committee.

The committee on "Cheoplasty" reported, which was received and adopted.

The committee to whom was referred for consideration, the style and construction of artificial dentures, denominated "*cheoplastic*," would respectfully submit the following report:

We have examined the subject as thoroughly as time and opportunity would permit.

We are unadvised in regard to the composition of the material of the base. It seems sufficiently dense, and it is stiff enough when formed into thin plates for all practical purposes.

It is not more readily bent or changed in form, than a swaged gold plate. It possesses, in our opinion, when properly constructed, quite as much, if not more strength, than a piece of work well constructed of gold.

We do not entertain a doubt about the durability of the work so far as strength is concerned.

In regard to the indestructibility of the metal, we have not much to say; because we do not know much about that. We are acquainted with the fact, however, that it has been in the mouths of various persons for several months, without any perceptible change. And it is worthy of notice, that some of those persons are competent to detect even the slightest change, either in the appearance of the metal, after it has been worn in the mouth, or by anything peculiar while in the mouth.

From what knowledge we have obtained in regard to this metal, we concur in the opinion, that it may be used in the majority of cases with impunity, and perhaps in all so far as destructibility is concerned.

There are two or three particulars in which it possesses excellences not to be found in other styles of work. There are no interstices or points for lodgment of food or other substances. In the construction of the pieces, the base is adapted to the teeth, and not the teeth to the base; and that adaptation is perfect. Consequently it is easily kept clean. Indeed, the same care required to keep the natural teeth clean, would preserve this in perfect condition. We consider the adaptation to the gums more perfect and more easily obtained, than by any other method. The metal neither contracts nor expands when passed from a low to a high temperature. It is cast to the model and runs very sharp, so that it makes a perfect counterpart of the model. Hence, a perfect fit is the result, if an accurate impression of the mouth has been taken. Any desired shape can be given to it for the more perfect restoration of the features. Any kind of teeth may be used in its construction. Those teeth in sections or blocks seem to be well adapted to it, and the ordinary gum or plain teeth may be used with facility; but teeth are now manufactured especially for this kind of work, so formed that it is impossible, after a piece is constructed, to remove them from the plate, without either cutting away or melting the plate, or breaking the teeth.

The facility with which it may be constructed is particularly worthy of notice; though we doubt not under the excitement of unanticipated success, impressions have been made on the minds of many, on this point, that will not be completely verified in the execution of the work. And we would suggest to those interested in the matter, that they should not make this point *too* prominent, though in *their* particular cases all they affirm may be correct. Yet we hesitate not to say, from our present knowledge of the work, that artificial dentures can be constructed by this mode with greater facility than by any other efficient mode in use. A tooth can be replaced, or any injury of the plate repaired without difficulty, and in a short time.

It has been objected that the work is too heavy; but it is not more objectionable in this respect than some other kinds, for instance, continuous gum work, or blocks on heavy gold plate.

There are some patients that will complain of any denture, however light, in the mouth. With such it is the *presence* of anything in the mouth, and not the weight that causes the objection. A heavy plate and perfect fit is far preferable to a light plate and an imperfect fit. It is also objected that the metal is not sufficiently indestructible. On this point we have nothing to say. Our experience in regard to this being very limited. On this particular, we rely mainly on those who have more experience, and who have for a much longer period and under more favorable opportunity, directed their attention in this channel.

All of which is respectfully submitted,

J. Taft,
H. N. Lewis,
S. Dunham,
A. M. Leslie,
H. McKellops,
} *Committee.*

The auditing committee on Treasurer's books made the following report which was adopted:

"The committee authorized to examine the Treasurer's report, have attended to that duty, and find his account correct."

H. McKellops,

S. Dunham,

} *Committee.*

The committee on dental patents, reported, which was received and adopted.

Whereas, The multiplication of dental patents in our country seems to be a source of reasonable apprehension lest the honor and standard of our profession be lowered. Therefore,

*Resolved*, 1st. That the principles of patents as usual in our profession of late, is unprofessional and not consistent with an honorable profession.

*Resolved*, 2d. That we do not consider it wrong for any member of our profession, who may invent a useful article in the manufacture of artificial structures or instruments, to patent the same, provided he confine his patent to the sale of the material at a reasonable charge, and not to the use of the same.

Dr. McKellops offered the following resolution:

Whereas, In consequence of great benefit enjoyed by the dental profession, from the peculiar manipulation of Dr. Arthur, in filling teeth, we feel it a duty and privilege to manifest our approbation of these benefits in some tangible form. Therefore,

*Resolved*, That a committee be appointed to prepare a testimonial of thanks, which shall be presented to Dr. Arthur by the Western Dental Society. Adopted.

Committee, Drs. McKellops, Taft and Allport, and on motion, the President was added to that committee.

Dr. Peebles offered the following:

*Resolved*, That a committee of three be appointed to present at an early period, the regular order of business, for the next annual meeting. Adopted.

Dr. Peebles,<br>

Dr. Perrine, } *Committee.*<br>

Dr. Blake,

The executive committee examined and presented Dr. G. Hanneman for membership, who, upon ballot, was declared duly elected.

The committee on fees and professional etiquette reported.

*Report of Committee on Fees and Professional Etiquette.*

*Resolved*, That the long experience and thorough course of study needful to render the dental practitioner competent to discharge the responsible duties of his profession, entitle him to a fair compensation for services rendered. That cheap dental operations, either surgical or mechanical, are dear and usually unsatisfactory to the patient, and tend to degrade the profession in the estimation of the community.

*Resolved*, That this society recommend the formation of local societies, for the purpose of regulating fees, and at the same time promoting harmony and good feeling among the members of the profession.

*Resolved*, That a dentist offering to travel out of his regular field of practice to perform operations in the field of a brother practitioner, for less than the usual fee, is discourteous and unprofessional. C. W. SPALDING.

The above report was adopted.

The President appointed Drs. Taft, Allport, Spalding, Branch, McKellops and Forbes, as delegates to the American Dental Convention.

On motion of Dr. Perrine, it was

*Resolved*, That when we adjourn, we do so to meet at Quincy, Ill., on the third Tuesday in July, 1858.

Adjourned to meet at 2½ P. M.

*Afternoon Session, 2½ P. M.*

Pursuant to adjournment, the meeting was called to order, President in the chair.

The minutes of the morning session were read and adopted.

The subject of "difficult dentition" was again called up, and elicited a very interesting discussion and a pledge from the members to aid in the acquisition of knowledge on this subject, by experiment, during the ensuing year.

On motion of Dr. E. Hale, jr., the subject of difficult dentition is made the special order of business for the next meeting.

The report of the committee on Prof. Arthur's plan of filling teeth was presented and adopted.

*Resolved*, That to Dr. Arthur, and he alone, the dental profession is under obligation for his liberality in laying before the profession the principle of using and welding together annealed gold, by the use of serrated pointed instruments, and that this society desire to express their thanks to him, for this one of the real improvements in the mode of operating.

H. McKELLOPS,
J. TAFT,
W. W. ALLPORT.

On motion of Dr. Allport, we now occupy the remainder of this session in free and promiscuous discussion, no member occupying more than ten minutes in one speech.

On motion of Dr. Allport, a committee of five was appointed to test local anæsthesia, and to report at the next meeting. Allport, McKellops, Dunham, Ferris and Porter were appointed on that committee.

The minutes were read, corrected and adopted.

On motion adjourned to meet in Quincy, Ill., July 22, 1858.

ISAIAH FORBES, President.

H. E. PEEBLES, Secretary.

P. S. After the meeting adjourned, a lot of specimen teeth and tin work, also a platinum case, also an old broken tin case, which had been used for a number of years, was received from Dr. W. G. Oliver, of Buffalo, N. Y., and presented by Dr. Perrine, who also read a lengthy letter from the same gentleman, to the various members who were still in the city.

P.

---

*Report of Discussions of the Third Regular Meeting of the Western Dental Society.*

*First Day—May* 20.

On taking the chair, Dr. Forbes, of St. Louis, in a few appropriate remarks, touched upon the importance of social intercourse among the members of the profession—said that our profession is called the twin sister of medicine—directed attention to the great responsibility resting upon practitioners of our art, considering it greater than that of other professions, not excepting either medicine or theology—alluded to the first meeting of the society, held in this city about a year ago, and called attention to the various methods of practice discussed at that meeting, mentioning the necessity of reviewing our operations for the past year, and of inquiring how far each member had been benefitted by these discussions.

Dr. Hale, Sen., of St. Louis, responds, thanking the society for the honor conferred upon him by electing him its first President.

*Afternoon Session.*—Subject: *What is the best method of arresting decay in teeth?*

Each member requested to describe his treatment of some particular case.

Dr. Hale, Sen., takes cavity in posterior surface of first bicuspid. After cleaning the cavity in a proper manner, and preparing gold in smoothly folded blocks, adapted in size to the size of the cavity, he first places a conical block in that portion of the cavity nearest the gum—then a block is introduced on each side, then one opposite the first, and then fills up the middle—condenses and finishes in the usual way.

Dr. Spalding, of St. Louis, takes a very simple case, that of a cavity in the grinding surface of molar. He first enlarges the orifice until the walls of the cavity are perpendicular, or nearly so; then slightly rounds the margin of the cavity, just enough to remove the sharp angles of the corners, and fills with cylinders, condensing the first one against the posterior wall, and so on till the cavity is full; then introduces a tapering point near the middle of the filling, condensing in every direction, and completes the plug by the introduction of a conical cylinder.

Dr. Allport, of Chicago, describes his treatment of cavity in approximal surface of superior central incisor. If the adjoining surfaces are parallel, would separate with file, and when possible, remove the entire cavity by filing. When the crowns are conical in shape, separates by pressing apart, and fills the cavity, avoiding the file as a means of separation when the teeth are to come into contact after the operation—uses cylinders in all approximal cavities.

Dr. Dunham, of St. Louis, exhibits his mode of filling cavity in anterior approximal surface of inferior lateral incisor—cavity large, extending nearly or quite down to cutting edge. Separates widely with file, fills with fold, using serrated instruments for packing—by their use is enabled to fill rapidly and to succeed in filling very shallow portions of the cavity, especially the point at or near the cutting edge of the tooth—thinks such fillings are not as hard as those composed of cylinders, but believes they are sufficiently dense, and are unusually successful—thinks more teeth are lost by the failure of fillings, and a consequent unwillingness on the part of patients to submit to a second operation, than from any other cause.

Dr. McKellops, of St. Louis, takes the case of a compound cavity in inferior second molar, involving both approximal surfaces, and extending entirely across the grinding surface. Makes a wide V shaped separation between first and second molars, and a narrow parallel one between the tooth involved and that standing behind it—first fills the body of the anterior cavity with cylinders, finishing at the top with annealed foil, used after Arthur's method, and extending the same towards the central cavity—then fills the body of the central cavity also with cylinders, connecting with the anterior by the use of annealed foil, as before—then fills the posterior cavity in a similar manner, allowing the projecting ends of the cylinder to rest against the wisdom tooth, and completes the filling by uniting the posterior and central portions by the use of annealed foil, as before—all projecting proportions are afterwards dressed off with a file.

Dr. Lewis, of Quincy, concurs with Dr. Allport, in the treatment of cavities in the incisor teeth, removes the cavity by the use of the file, when that can be done, the proper method has been already described by others.

Dr. Blake, of St. Louis—The subject before the society for discussion is the best method of filling an incisor tooth.

The case I present is a lateral, badly decayed on the front approximal surface, the lower portion having broken away.

On examination, I find the only part of the tooth which can be retained is from the center of the cutting edge to the surface of the tooth where it enters the gum.

As teeth of this description may be preserved and restored to their natural shape, I will briefly give the method I adopt in their treatment.

Before I proceed, however, I wish to remark that I do not in all cases advise filling teeth that are thus decayed. Formerly, all of this class and condition were made to give place to artificial work; but now, with the improvements in our art, it is otherwise. I would also say that I am indebted to Dr. Arthur, of Philadelphia, and to Dr. Nichols, a member of this association, for important suggestions on this subject.

I will now suppose a patient seated in the chair, with an incisor as above described. After examining it and its neighbors, if I determine on its preservation, I lay all my plans, then proceed with the operation; first, with a file I make the edges even and smooth, against which I wish to build my filling, then with excavators and drills, carefully clean and prepare the cavity. If the nerve is exposed, I treat and remove it, then fill the fang, which leaves a good attachment for the plug; but if it is possible to preserve the nerve, I in all cases do so. If I do not attach to the filling of the fang, I usually make two places of attachment, one near the upper portion of the cavity, and the other as near the lower as the strength of the tooth will permit. Having thus prepared the cavity, I proceed to anneal the gold.

Have generally used Dunlevy's for this purpose, but recently have been using foil manufactured by our worthy Secretary, Dr. Leslie. I divide a sheet into three equal parts, and fold so as to make my strips about one-fourth of an inch in width. I cut these in pieces about five-eighths of an inch in length. I now place these pieces, as many as convenient, without touching each other, on a very thin piece of platina, and heat over a spirit lamp, using a blowpipe, so as to raise a white heat.

Having a sufficient quantity prepared and all things ready, I direct the patient to take as easy a position as possible, telling him it will require some time to complete the operation. I proceed to dry the cavity, using prepared cotton.

I always use serrated instruments for these plugs, but seldom have more than three points. I generally take three pieces of gold to commence, but after making the attachment to the tooth, use only one piece at a time, carefully and thoroughly condensing as I proceed.

In this manner, if kept dry, we may build out to any extent we wish. I build to a little more than the full size of the tooth, then dress down with files to its proper size, giving it as natural a shape as possible. I next burnish, then stone down and burnish again. It is now ready for fine pulverized pumice stone, which I use with soft wood, also with tape. I again burnish lightly, which gives a high finish. As this, however, would strike unfavorably the eye of the beholder, I touch it over carefully with pumice. This completes the operation.

Dr. Barron, of St. Louis, exhibits his treatment of compound cavity in superior central incisor; uniting both approximal surfaces, extending across the cutting edge, and accompanied by the loss of the lower portion of the crown to the extent of one-third of its whole length. First, fills with upright cylinders, extending as far down as the enamel is sound, reserving a small point in one corner, where he commences the use of crystal gold, with this substance he builds down, and thus restores the tooth to its original form and size, gold supplying the place of the lost dentine.

Dr. Silvers, of St. Louis, takes cavity in grinding surface of molar. Prepares cavity same as Dr. Spalding; fills with rough pellets, thinks them more adhesive than cylinders, and more readily compressed. In condensing, opens clear down to bottom of cavity; thinks a wedge or cone, placed in the middle, would be liable to be driven out by an antagonizing tooth, when it strikes the side of the plug; has never used cylinders, and thinks never shall.

Dr. Sturgis, of Quincy, takes a case of a dishing cavity locating in one cusp of grinding surface of molar. Cuts small grooves in sides of cavity, and fills with foil in rolls, sometimes uses the fold.

Dr. Peebles, of St. Louis, heard from Dr. Hale last year about filling fangs with plaster of paris, tried this substance in fangs of lower molars in the case of a poor patient, in one tooth found it difficult to introduce, in the other, was introduced easily—filled crowns with gold, successful so far.

Dr. Perrine, of St. Louis, gives a case of use of plaster in filling fang of lower bicuspid. After five months, tooth became troublesome—on removing gold filling, found the plaster soft, unfavorable result.

Dr. P. also describes case of large cavity in superior cuspid, involving death of nerve and the loss of posterior portion of crown—lower cuspid, owing to loss of back teeth, striking into the cavity—filled fang with foil, then completes the operation by building up against the thin front wall of enamel, after Arthur's plan of using annealed gold. Feels more confident of success in the use of foil in this manner than in the use of crystal gold.

Dr. Hubbard, of Quincy, takes a central cavity in grinding surface of molar, with four diverging lines of decay. First opens central cavity with drill, then enlarges the diverging lines, fills first the lateral lines, then the posterior, then the central cavity, finishing with the anterior radiating line.

Thinks it not necessary to remove every portion of decayed matter from the bottom of the cavity, especially if in so doing, the nerve would be uncovered—thinks the formation of new osseous matter will occur in young persons, in the course of six or eight weeks. Sometimes uses gutta percha over exposed nerves; believes we destroy too many nerves, and often do so when it is not necessary. Does not succeed in more than one-half the cases in which he has practiced filling fangs.

Dr. Porter, of Michigan, exhibits his treatment of cavity in approximal surface of superior incisor, accompanied by loss of corner on cutting edge; fills partly with foil, and partly with crystal gold; rolls foil into rope, then unrolls and rolls again, to produce increased roughness, uses instruments with serrated points, allows the foil in packing to rest against the adjoining tooth, builds down with crystal gold, to restere the broken corner to original shape.

Dr. Levett, of St. Louis, takes a case similar to the last.

Uses the fold or strip, and builds out with foil, annealed and used in the manner recommended by Dr. Arthur, of Philadelphia.

Dr. Bull, of Lasalle, treats sensitive dentine with solution of gutta percha, after a few days removes gutta percha, and fills with foil twisted into a rope.

Dr. Taft, of the Ohio Dental College, remarked, when a case is presented, he takes into consideration the constitutional tendencies and temperature of the patient, also, the strength of the vital forces, the liability to irritation, and the probable influence of remedial treatment.

This diagnosis is made up from external indications, also by these forms an opinion as to the character of the teeth. These indications are found in the development and condition of the various tissues, the color and tone of the skin, etc.; thus we can know the general character of the teeth before a special examination is made. We cannot operate alike on all teeth. Some would be destroyed by the same operation that would save others, owing to constitutional differences. These differences are too frequently disregarded, even by those whom we consider good operators.

We will take, for description, a cavity of the anterior surface of superior molar. The decay occupies almost the entire approximal surface. The first step is the special examination, to ascertain the nature and extent of the decay, the form of the cavity, and the strength of the walls. Then separate. There are two methods of accomplishing this—by pressure, and by cutting. The latter only is applicable in this class of cases. He uses the chisel instead of the file in cutting—can cut more rapidly by this means, and with less annoyance to the patient. Smooths the cut surface with a fine file.

The next step is to remove all the decay. Though in some cases, where the nerve would be exposed, it may be admissible to permit a small portion of partially decomposed bone to remain for a covering, but in no case should we leave even the slightest portion of decaying bone about the orifice of the cavity, or even upon the walls. Then give the cavity such form as will certainly retain the filling. It is very seldom the case that when the decay is removed, the cavity is of the proper shape; and hence, the formation of the cavity constitutes another step.

The cervical wall of the cavity should be a little inclined inwards, the lateral and crown walls, at about a right angle.

For drying the cavity, use prepared cotton, sometimes blotting paper; after this, throw in a jet of warm air, drying the cavity to whiteness.

In filling either with crystal gold or foil in blocks, commence the filling at the cervical wall of the cavity, filling down two-thirds to the crown wall, consolidating perfectly to the lateral walls. Then fill to the crown wall of the cavity, filling up to the portion first introduced, terminating the introduction of the gold somewhere near the center of the filling.

In the application of force for the consolidation of fillings, it should be a general rule to apply the pressure as nearly on a direct line with the axis of the tooth as possible. Consolidate the surface perfectly, and finish with the file, pumice, Scotch stone, and buff or burnish.

In these decays, the crown wall is often broken away, leaving the two lateral walls standing, with strong projections or angles. There are two methods of operating in such cases. First, to cut down these angles back as far as the crown wall is broken away. That makes the surface at the lateral borders diagonal with the axis of the tooth, and the surface of the filling will partake of the same inclination. And second, the angles, if firm, may be left standing, and the filling introduced and brought out flush with the angles; thus forming the filling with an approximal and a crown surface. The angle formed by the filling should not be too sharp.

On the subject of building out fillings, Dr. Allport said, that from some things that had been said, he feared there was some misapprehension as to his views on this point. He did not practice it generally, but would resort to it only occasionally; believes that more was being done in that way just at this time than would be done a few years hence. If practitioners were were not careful, they would ride this hobby too hard.

Dr. Spalding said he did not like the practice, excepting in very rare cases—would hardly resort to it at all in back teeth, and very rarely in front ones.

*Thursday Morning.*—Subject: *The Causes and Treatment of Diseased Gums.*

Dr. Spalding said he would pass over, for the present, the first part of the question, relating to causes, and confine himself to that portion relating to treatment only. In the first place, he would, of course, carefully and thoroughly remove all accumulations of foreign matter, repeating the operation as often as might be necessary, at the same time also excising an excessive prurient growth of the gums. The next step would be the employment of such means as would serve to assist nature in restoring a healthful condition to the diseased parts. He had long ago laid aside the use of astringents in cases of this kind, and relied altogether upon the use of stimulants. In the local treatment demanded in these cases, it had been his object to rouse up and stimulate the action of the secretory and absorbent vessels, and he had met with better success than formerly, when employing astringents. He relied chiefly upon the different alkalies, and would be guided in his selection mainly by the taste, choosing those which were found the least unpleasant. Directed the patient to make free use of the brush, using as firm a one as the gums would bear—considered the friction of the brush highly useful in effecting a cure.

Dr. Allport does not rely much on medicines. Carefully removes all accumulation, scarifies freely, and rubs with a soft brush to promote depletion—subsequently directs the use of Prof. Westcott's recipe, consisting of equal parts nitrate of potassa and carbonate of soda, dissolved in a strong decoction of sage, sweetened to improve the taste, to be used after each meal.

Dr. Dunham: Sometimes finds it necessary to remove teeth as well as tartar. Employs scarification and also excision. Sometimes uses nitrate of silver, but always avoids a resort to such active remedies, if possible. As a wash, relies mainly on tinc. myrrh, made with rectified whiskey instead of alcohol; also uses carbonate of soda and nitrate of potassa—knows of nothing as good as simple tinc. myrrh, which is astringent, stimulant and antiseptic.

Dr. Hale, Sen., said, the principal causes were neglect, want of cleanliness, and consequent accumulation of tartar. After the necessary cleaning, treats with astringent washes and powders, such as myrrh, tannin, etc. Where suppuration of the gums exists, finds the case difficult to treat with success; regards periostitis as the primary disease in these last cases, and the suppuration of the gums as secondary; thinks friction and cleanliness are much more important than the use of astringents in all cases, regarding the employment of friction as indispensable to a cure.

Dr. Blake considers tartar accumulations as a leading cause, carefully removes, then scarifies, and also uses artificial leeches as an additional means of depletion—uses tinc. myrrh, with the addition of a little creosote—thinks astringents valuable.

Dr. Phillips, of Mo.—Cleanses. Scarifies freely, to produce depletion, and sometimes employs astringents, not often.

Dr. Sturgis. Thinks diseases of the gums hereditary—irregularity also a cause; uses myrrh, but does not generally find its use followed by a permanent cure.

Dr. Silvers mentions a case in which he employed mechanical support to retain the teeth in their places—treated with nut galls, sulph. ether, and creosote.

Dr. Levett, after cleaning and scarifying, uses borax and honey, also astringents—thinks diseases of the gums hereditary, especially where there is a venereal taint.

Dr. Peebles. Is an inquirer. Has not yet satisfied himself in regard to his own treatment; has used many remedies recommended by others, but has not found them effectual. Has found long "gourd-seed" teeth more subject to diseases of the gums and alveoli. Can not cure such cases short of extraction.

Dr. Perrine. Has nothing new or additional to offer; has a case of necrosis of alveoli. Has failed with astringents, and now proposes to adopt the use of stimulants.

Dr. Hubbard. Nothing to add, the ground having been already gone over—relies very much upon depletion by scarification—has used astringents and alkalies—has met with success in the use of solution of nitrate of silver—meets with cases that he cannot manage—thinks nitrate of silver has a direct action on the mucous membranes.

Dr. Porter. Nothing to add; uses in extreme cases nitrate of silver, in ordinary cases, recommends use of soap, also uses astringents, and scarifies.

Dr. Branch, of Galena, uses both astringents and stimulants. If the teeth are of the long gourd-seed variety, thinks the leverage greater in mastication, such teeth having also openings between them near the gum. Food there accumulates, and by mechanical pressure on the gums, produces absorption and disease. Reports a case of removal of tumor from the gum by ligature, producing a cure. In ordinary cases, depends chiefly on friction from the use of the brush; directs the employment of a hard brush, to produce bleeding; objects to excision, preferring to wait the operation of nature, and in cases of suppuration of the margin of the gum, advises extraction of the teeth; for acute periostitis, prescribes spirits of nitre, saturated with calcined alum.

Dr. Taft remarked, there are cases that do not yield to any ordinary treatment. Such cases are usually influenced by both local and constitutional causes. When the latter exists, constitutional treatment is indicated, but when it is the result solely of local causes, then local treatment only is indicated. This consists of excision occasionally, depletion, and counter-irritation by scarifying. The medicinal treatment consists of astringents, stimulants and tonics. When the vital tone is low, slight local causes will produce disease. In some instances, the most heroic treatment is indicated—in others, touch lightly. Constitutional treatment may require the aid of a physician. He referred to a case. A lady, 22 years of age, good constitution. Found considerable constitutional disturbance and lassitude; gums much enlarged, covering roots and some sound crowns. The free margin of the gums presented a rounded mass of one-half to three-fourths of an inch in diameter. The patient used compound extract of colocynth. Excised the enlarged portion with the bistoury, passing it entirely around the arch, at about the line of the gum margin in health. Removed all the roots and some of the teeth, and on the second day afterward, removes any of the excrescences that have been overlooked; used astringent wash for two or three days, then used nitrate of silver in substance. The mouth entirely restored in five or six weeks. Prefers excision to waiting the slow process of absorption.

Dr. Forbes says there are three principal causes, local, constitutional and scrofulous. Cleanses thoroughly—is not particular to avoid laceration. Excises with pointed scissors in some cases. Prescribes calcined alum and pulv. cinnamon. Uses creosote, either pure or diluted, in periostitis; also uses calcined alum and creosote; uses tinct. of myrrh in all cases.

Dr. Hale, sr. treats common cases with powdered nut gall or tannin, followed by nitrate of silver in solution—generally successful in cases of children.

*Afternoon Session.*—(Miscellaneous discussion not reported.)

*Friday Morning.*

*Discussion on Report of Committee on Patents, &c.*—Dr. Spalding objects to the phraseology of the report.

Dr. Allport is opposed to patenting anything beyond instruments and other articles of manufacture. If instruments or materials on which a patent has been obtained are thrown into the market and freely sold to any and all who desire to purchase, he has no objection to a patent—but he is strenuously opposed to the sale of office rights in any case whatever.

Dr. Branch offers a substitute changing the phraseology of the pending resolution.

The whole report is recommitted, and the committee retire.

Report on Cheoplasty by Dr. Taft, Chairman, presented and adopted without debate.

Resolutions on patents are again reported and discussion resumed.

Dr. Spalding was not prepared to oppose the *entire* principle of patenting, as seemed to be contemplated in the first resolutions reported by the committee—did not feel willing to go to that extreme. The government had instituted the patent system for the purpose of protecting inventors in their rights. Men who had expended their time and money in perfecting improvements in the mechanic and other arts, could only in this way secure to themselves the results of their own labors and expenditures. He considered copy-righting as only a part of the same general system, extending protection to authors as well as inventors, and if instruments and other articles of manufacture could be sold in the unrestricted way in which books were, he had no objections to a patent. He was willing that inventors should exercise the same control over the manufacture of patented articles that authors did over the publication of copyrighted books, and that both inventors and authors should look wholly to the sale of their productions for remuneration for their labors. He agreed with the views held by Dr. Allport; indeed, Dr. A. had exactly expressed his mind on this subject. Neither did he approve the course taken by some, of shielding themselves under the name of others. If it was right for a professional man to hold any interest in, or control over a patent, it was not only right, but in his opinion far more honorable for him to take a frank, open and straight-forward course and hold it in his own name. But when any man belonging to a liberal profession claimed the right of patenting any mode of practice, and proposed, directly or indirectly to peddle out office or territorial rights, he for one, would go as far as any body towards heaping upon such a custom all the censure which it merits at our hands. He had drawn a distinction in this matter of patents, which he conceived to be the correct one, and he was glad that so many whose opinions he valued, were of the same way of thinking with himself.

Dr. Barron said that the second resolution offered by the committee qualified the first, and as Chairman of that committee, he was willing to so modify the first as to meet the views of members. For himself, he was strongly opposed to the growing custom of resorting to patents, and if some check was not put upon it, it would soon be the case that a dentist could not go into his office and commence any operation without first buying a patent instrument, and then purchasing the right to use it.

Dr. Branch is not opposed to the custom of patenting. Thinks if it is right to patent at all, it is right in one profession as well as another; thinks we should fix upon some rule for the guide of patentees or of inventors, and should say that the principle is right or wrong.

Dr. Allport thinks the resolution before us, as it now stands, only condemns the course pursued by some, and does not cover the whole principle of patenting; thinks that no member of a liberal profession has a right to first patent an instrument or material and then patent its use. He alluded to the very excellent dental chair invented and patented by his friend, Dr. Perkins. When a man bought one of Dr. P's chairs, the right of use went with the chair; and, as in the case with a book, it might be sold and resold, and still the last owner had just as much right to its use as the first. The example of Dr. Perkins was worthy of all imitation. He had not only constructed the best chair ever yet made, but he had placed it within the reach of every dentist, so that all might enjoy the benefit resulting from its use.

*Friday*, 3 *P. M.*

*Difficult Dentition.*—Dr. Spalding spoke of the importance of the subject under consideration; thought that a large per cent. of the mortality among infants could be fairly referred to this cause; thought physicians have not given the subject that careful attention to which it was entitled, owing, no doubt, to the wide range of subjects which necessarily come within the scope of medical practice. Said the subject fairly came within the province of the dentist, and earnestly hoped that the members here present would take up the matter in good earnest, was himself comparatively ignorant, but would like to hear from Dr. Taft, who has long studied the subject, and could, he believed, impart much important information.

Dr. Taft said there are various causes of difficult dentitions. In some cases there seems to be a want of assimilating powers; in other cases an imperfect supply of the proper material. This is manifested by a deficiency in the development of the osseous structure, and sometimes extends to the muscular and other organic tissues also. When the deficiency is general, it falls within the province of the physician. This deficiency extends to the teeth and affects them as well as other bony structures. Usually there is a deficiency of bone phosphate. To supply this, the administration of phosphate of lime has been resorted to, and with very marked success.

It may be given to the child, or to the mother while the child is nursing, or to both; this influences the eruption of the teeth, by causing a more rapid development. No difficulty arises from its exhibition; it is readily appropriated, but in different degrees. Have gone a little farther back than this. A mother had three children, and in each case their primary teeth decayed very early; indeed, they seemed to be scarcely through the gums till they began to decay.

The mother used bone phosphate for four months previous to the birth of the child, and while the child was nursing. That child had firm, well organized teeth, that seem as though they will resist decay till the eruption of the permanent ones.

Related a case of the rapid restoration of a child 16 months old, that was reduced to an extreme degree of emaciation and debility. The cause seemed to be difficult dentition. General treatment was continued much as before, and the administration of bone phosphate adopted at once. The child immediately began to improve, and in two months seemed to be entirely restored, cutting six teeth soon after beginning the use of the phoshpate; gave child from two to six grs. per day, in two portions; when administered to the mother, from 6 to 15 grs. per day, in two portions.

Its use is not indicated when the fontanelle closes readily, and there is no apparent want of bone material.

